# Circulating extracellular vesicle-containing microRNAs reveal potential pathogenesis of Alzheimer’s disease

**DOI:** 10.3389/fncel.2022.955511

**Published:** 2022-10-20

**Authors:** Yi Wang, Ping Yuan, Lu Ding, Jie Zhu, Xinrui Qi, Yanyan Zhang, Yunxia Li, Xiaohuan Xia, Jialin C. Zheng

**Affiliations:** ^1^Translational Research Center, Shanghai Yangzhi Rehabilitation Hospital Affiliated to Tongji University School of Medicine, Shanghai, China; ^2^Department of Cardio-Pulmonary Circulation, School of Medicine, Shanghai Pulmonary Hospital, Tongji University, Shanghai, China; ^3^Center for Translational Neurodegeneration and Regenerative Therapy, Tongji Hospital Affiliated to Tongji University School of Medicine, Shanghai, China; ^4^Center for Translational Neurodegeneration and Regenerative Therapy, Shanghai Tenth People’s Hospital Affiliated to Tongji University School of Medicine, Shanghai, China; ^5^Department of Neurology, Tongji Hospital Affiliated to Tongji University School of Medicine, Shanghai, China; ^6^Translational Research Institute of Brain and Brain-Like Intelligence, Shanghai Fourth People’s Hospital Affiliated to Tongji University School of Medicine, Shanghai, China; ^7^Shanghai Frontiers Science Center of Nanocatalytic Medicine, Tongji University, Shanghai, China; ^8^Collaborative Innovation Center for Brain Science, Tongji University, Shanghai, China

**Keywords:** Exosome, miRNA, Alzheimer’s disease, neurodegeneration, serum

## Abstract

The pathogenesis of Alzheimer’s disease (AD) remains unknown till today, hindering the research and development of AD therapeutics and diagnostics. Circulating extracellular vesicles (EVs) can be utilized as a new window to spy upon AD pathogenesis. Altered microRNA profiles were noted in both the cerebrospinal fluid (CSF)- and blood-isolated EVs of AD patients, implying the outstanding potential of circulating EV-containing miRNAs (CEmiRs) to serve as important regulators in AD pathogenesis. Although several CEmiRs were found to play a part in AD, the association of globally altered miRNA profiles in patients’ serum-derived EVs with AD pathogenesis remains unclear. In this study, we first investigated the miRNA profile in serum-derived EVs from AD, mild cognitive impairment (MCI) patients, and healthy individuals. We observed differential expression patterns of CEmiRs and classified them into 10 clusters. We identified the predicted targets of these differentially expressed CEmiRs (DECEmiRs) and analyzed their biological functions and interactions. Our study revealed the temporal regulation of complex and precise signaling networks on AD pathogenesis, shedding light on the development of novel therapeutic strategies, including multi-target drug combination for AD treatment.

## Introduction

Alzheimer’s disease (AD) is the most common chronic and progressive neurodegenerative disease among people over 65 years of age. It is increasingly prevalent given that the global population is aging. There is neither cure nor effective early diagnostics for AD due to the fact that most patients are only diagnosed after typical symptoms like memory loss show up, which unfortunately lag 10–20 years to the first occurrence of pathological changes in the brain. The pathogenesis of AD remains unknown till today, hindering the research and development of AD therapeutics and diagnostics.

Extracellular vesicles (EVs) are small bilipid layer-enclosed EVs that can be found in tissues and biological fluids (Xia et al., 2019). As key intercellular communicators, EVs mediate the pathogenesis of various neurological disorders, potentially through transferring pathogenic cargos to target cells (Xia et al., 2019). EVs have been reported to contain AD-related molecules including Aβ (Jia et al., 2019) and Tau proteins (Wang et al., 2017), and to facilitate the spreading of these pathogenic molecules in the brain (Dinkins et al., 2014; Asai et al., 2015). More importantly, EVs derived from brain cells can be found in the blood due to their blood–brain barrier penetration capacity (Fiandaca et al., 2015). Thus, circulating EVs may be utilized as a new window to spy upon AD pathogenesis.

MicroRNAs (miRNAs) are a type of phylogenetically conserved small non-coding RNAs (18∼24 nucleotides) that are enclosed and delivered by EVs (Ambros, 2004; Bartel, 2004; Xia et al., 2019). Through modulating the expression of complementary messenger RNAs (mRNAs), miRNAs regulate a wide variety of physiological and pathological processes like cell fate determination, cell death, and immune responses (Bartel, 2004; Noren Hooten et al., 2013; Zhang et al., 2015). Inspiringly, altered microRNA profiles were noted in both the cerebrospinal fluid (CSF)- and blood-isolated EVs of AD patients, which exhibit a greater potential to serve as new biomarkers in the diagnosis of AD (Cheng et al., 2015; Gui et al., 2015; Xia et al., 2019). Moreover, certain differentially expressed miRNAs, such as miR-193, in AD patient serum-derived EVs have been demonstrated to target *APP* mRNAs and inhibit APP expression *in vitro* (Liu et al., 2014). However, the association of globally altered miRNA profiles with AD pathogenesis remains unclear. In this study, we first investigated the miRNA profile of serum-derived EVs from AD, mild cognitive impairment (MCI) patients, and healthy individuals. We observed differential expression patterns of circulating EV-containing miRNAs (CEmiRs) and classified them into 10 clusters. We identified the predicted targets of these differentially expressed circulating EV-containing miRNAs (DECEmiRs), and analyzed their biological functions and interactions. Our study revealed the temporal regulation of complex and precise signaling networks on AD pathogenesis, facilitating the development of AD diagnostics and therapeutics.

## Materials and methods

### Study population

The design of the present study was approved by the ethics committee of Tongji Hospital affiliated to Tongji University School of Medicine (Shanghai, China), and written informed consent was obtained from all participants. A total of three age-matched non-dementia controls (mean age 70.0 ± 7.1 years), three AD patients (mean age 67.3 ± 4.7 years), and three MCI patients (mean age 69.2 ± 5.9 years) at their first clinic visit were enrolled for this study. The detailed baseline characteristics of study participants are provided in Supplementary Table 1. The inclusion criteria were as follows: (1) patients fulfilled the diagnostic criteria of NINCDS-ADRDA; (2) the age of the patients ranged from 60 to 80 years; (3) Mini-Mental State Examination (MMSE) and Montreal Cognitive Assessment (MoCA) scores of 0–25; and (4) cranial computed tomography or magnetic resonance showed hippocampus atrophy. The exclusion criteria were as follows: patients with severe physical illness; serious diseases of the heart, liver, kidney, and hematopoietic system; past or present cerebral hemorrhage; traumatic brain injury; and other neurological or psychiatric diseases.

### Sample collection

Ten milliliters of whole blood was collected from each donor. The serum was separated by centrifugation at 3,000 g for 10 min in room temperature, followed by centrifugation at 12,000 g for 5 min at 4^°^C. The samples were used for EV collection immediately.

### Collection of extracellular vesicles from human serum

The EVs in the serum were isolated using the ExoQuick Exosome Precipitation kit (System Biosciences, Palo Alto, CA, USA) according to the manufacturer’s instructions. Briefly, 500 μl of serum was mixed with 125 μl of ExoQuick Exosome Precipitation Solution, kept upright, and incubated for 30 min at 4^°^C without rotation. The mixture was centrifuged at 1,500 g for 30 min at 4^°^C to spin EVs down. EVs were resuspended in PBS and stored at –80^°^C until required.

### Nanoparticle tracking analysis

Nanoparticle tracking analysis (NTA) was performed as previously described (Ding et al., 2022). Briefly, 1 ml of diluted EV suspension was used for NanoSight analysis. NTA was assessed on the NanoSight NS300 system (Malvern Instruments, UK) with an sCMOS camera. NTA was carried out in the conditions at 25^°^C, 1 cP viscosity, 25 s per capture frame, and 60 s measurement time. Three individual measurements were performed to determine the size of EVs.

### Western blotting

Western blotting was carried out as previously described (Gao et al., 2020). EVs were lysed in RIPA lysis and extraction buffer (Thermo Fisher Scientific, Waltham, MA, USA) containing a protease inhibitor cocktail (Sigma, St. Louis, MO, USA). Protein concentrations were measured by BCA Protein Assay Kit (Thermo Fisher Scientific, Waltham, MA, USA). Proteins (20 mg) from EV lysates were loaded on sodium dodecyl sulfate-polyacrylamide gel electrophoresis (SDS-PAGE) gels and then electrotransferred to polyvinylidene fluoride membranes (Bio-Rad, Hercules, CA, USA). The membranes were blocked in freshly prepared 8% non-fat milk diluted in PBS and incubated with primary antibodies for CD9 (rabbit, absin, cat# abs102480, 1:1,000), Flottlin2 (rabbit, cell signal, cat# cst3436S; 1:1,000), and CD63 (rabbit, Abcam, cat# ab134045, 1:1,000) overnight at 4^°^C followed by a secondary anti-rabbit or anti-mouse antibody (Cell Signaling Technologies, 1:10,000) incubation. Antigen-antibody complexes were visualized using a Pierce ECL Western Blotting Substrate (Thermo Fisher Scientific, Waltham, MA, USA). The films were scanned with a CanonScan 9950F scanner, and the band densities were digitally measured using ImageJ software.

### MicroRNA microarray

Total RNA was extracted from EVs, and 1 ng of total RNA per sample was used as input material for the small RNA library. Sequencing libraries were generated using NEBNext^®^ Multiplex Small RNA Library Prep Set for Illumina^®^ (NEB). The clustering of the index-coded samples was performed on a cBot Cluster Generation System using TruSeq SR Cluster Kit v3-cBot-HS (Illumina). After cluster generation, the library preparations were sequenced on an Illumina Hiseq 2500/2000 platform and 50-bp single-end reads were generated. Raw data (raw reads) of fastq format were first processed through custom Perl and Python scripts for quality control. The small RNA tags were mapped to the reference sequence by Bowtie without mismatch to analyze their expression and distribution on the reference. Mapped small RNA tags were used to look for known miRNA. miRBase 20.0 was used as a reference for known miRNA, and miRDeep2 and sRNA-tools-cli were used to obtain novel miRNAs and draw the secondary structures, respectively. miRNA expression levels were estimated by TPM (transcript per million). Comparisons between different stages were made to identify significantly different DECEmiRs (fold change ≥ 1.5 or ≤ 0.5, *p* < 0.05).

### Bioinformatics analysis

To classify DECEmiRs with similar temporal expression patterns at different stages of AD, a set of unique model expression tendencies was identified in accordance with different signal density change tendencies of miRNAs in the stage course. The raw expression values were converted into log_2_ ratio. Using a mathematical fuzzy clustering strategy by Short Time-series Expression Miner (STEM) version 1.3.12 software, several unique profiles were defined. STEM was run using the “normalize data” option, with all other settings set to the defaults. The expression model profiles were related to the actual or the expected number of miRNAs assigned to each model profile.

The predicted target mRNAs of DECEmiRs were obtained from TargetScan.^1^ The enrichment of gene ontology (GO) term and KEGG pathways in predicted target mRNAs were analyzed using DAVID online tools.^2^ GO terms corresponding to biological process, cellular component, and molecular function were selected. The enrichment of GO terms and KEGG pathways was determined by the *p*-value from DAVID online tool analysis. The *p*-values were determined by a modified Fisher’s exact test, adjusted by the Benjamini-Hochberg method.

### Statistical analyses

All results are the means of at least three independent experiments ± s.d. Data from two groups were evaluated statistically by a two-tailed, paired, or unpaired Student’s *t-*test. Significance was considered when *p* < 0.05.

## Results

### The profiles of microRNA within extracellular vesicles are significantly altered in sera of mild cognitive impairment and Alzheimer’s disease patients

For elucidating the expression patterns of CEmiRs in the sera of MCI and AD patients, we first characterized EVs through NTA and Western blotting assays. NTA results demonstrated that the size of EVs among the groups was between 100 and 200 nm, matching the typical size distribution of EVs (Supplementary Figure 1). Western blotting also displayed abundant expression of EV-positive markers like CD9, Flotillin2, and CD63 in EVs among groups (Supplementary Figure 2). These results confirmed that the vesicles isolated from the serum were EVs.

**FIGURE 1:**
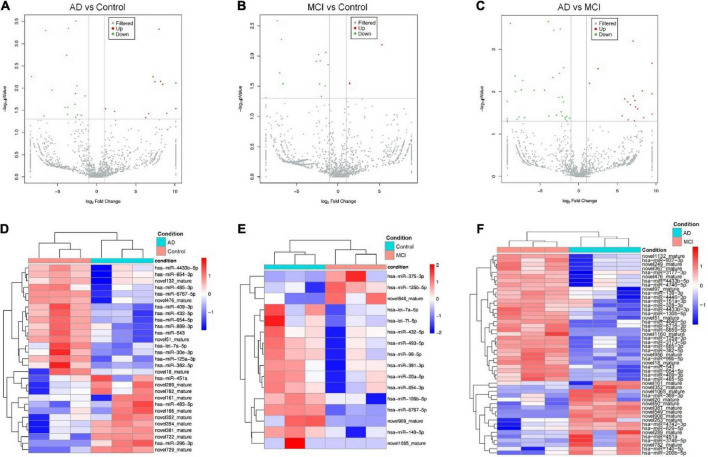
Circulating exosomal miRNA expression profiles at different progression stages of AD. **(A–C)** The volcano plots of CEmiRs in AD vs. Control comparison **(A)**, MCI vs. Control comparison **(B)**, and AD vs. MCI comparison **(C)**. Dots in the volcano plot represent DECEmiRs. Values on X-axis refer to relative fold (log_2_) between the expression levels of a given miRNA in EVs. Values on Y-axis refer to *p*-value (log_10_) in each comparison. **(D–F)** Heatmap representation of the mean fold change of DECEmiRs in AD vs. Control comparison **(D)**, MCI vs. Control comparison **(E)**, and AD vs. MCI comparison **(F)**. Rows stand for DECEmiRs. Each entry of the grid refers to relative fold (log_2_) between the expression level of a given miRNA in EVs. The color of each entry is determined by the value of that fold difference, ranging from blue (negative values) to red (positive values).

We next profiled miRNAs isolated from serum-derived EVs of healthy individuals, MCI, and AD patients using the microarray approach. A total of 727 known miRNAs and 1,172 novel ones were detected by the miRNA microarray analysis. All identified CEmiRs were then plotted into volcano plots to display DECEmiRs. We identified 12 upregulated and 17 downregulated miRNAs between AD and control groups (Figure 1A), 3 upregulated and 13 downregulated miRNAs between MCI and control groups (Figure 1B), and 18 upregulated and 31 downregulated miRNAs between AD and MCI groups (Figure 1C). To better display upregulated and downregulated DECEmiRs, heatmaps were generated based on the expression levels of each DECEmiRs between AD and control groups (Figure 1D), MCI and control groups (Figure 1E), and MCI and AD groups (Figure 1F). Interestingly, among all the three groups of comparisons, the highest numbers of miRNAs were identified in the AD vs. MCI comparison. It indicates that the progression of AD is a highly dynamic process that may be associated with diverse miRNAs, and the tremendous pathological changes between the MCI induced by neural circuit dysfunction and AD characterized by neuronal loss-mediated brain atrophy, suggesting the importance to include the MCI stage in investigations that aim to clarify AD pathogenesis.

**FIGURE 2:**
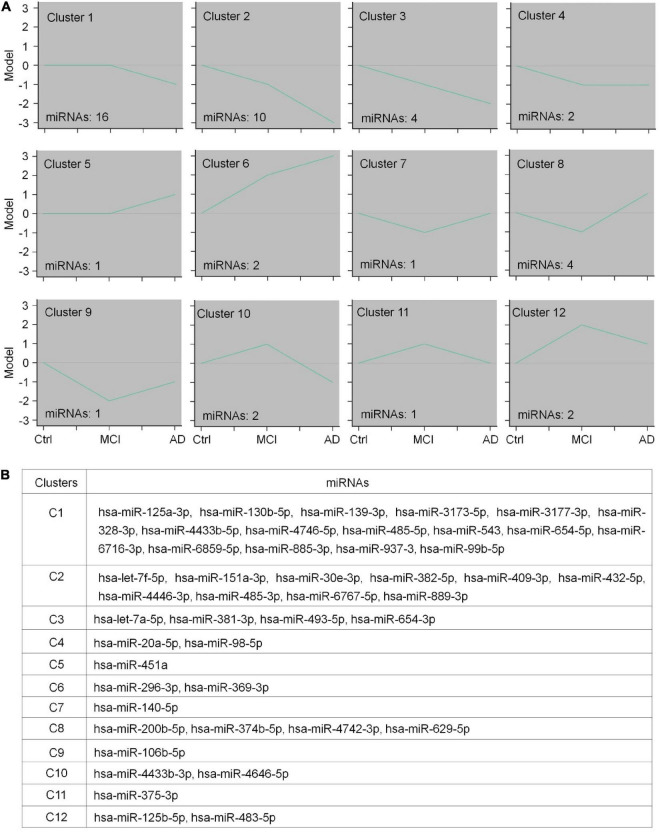
The expression patterns of differentially expressed circulating EV-containing miRNAs (DECEmiRs). **(A)** The expression profiles of DECEmiRs during AD progression were classified into 12 clusters using a mathematical fuzzy clustering strategy by STEM software. The green lines in the square indicate the overall gene expression trends. The number on the upper left of the small square indicates the cluster number; the number on the lower left of the small square indicates the DECEmiR number. Y-axis refers to relative expression levels of miRNAs, and X-axis indicates the stages of AD (controls, MCI, and AD). **(B)** Member DECEmiRs in the identified STEM clusters.

To identify CEmiRs involved in different stages of disease progression based on their dynamic expression profile, we used the STEM algorithm to analyze our microarray data. In this analysis, we only included named (confirmed) miRNAs and excluded all predicted miRNAs that are not commented on by the miRBase database.^3^ Twelve clusters were created to represent the temporal expression patterns for healthy control, MCI patients, and AD patients (Figures 2A,B). As shown in Figure 2A, the expression of miRNAs in clusters 1–4 showed a trend of decrease with disease progression. To be more specific, the expression of CEmiRs in cluster 1 significantly decreased in AD compared with that of MCI and healthy controls. The expression of CEmiRs in clusters 2 and 3 was consistently downregulated during the progression of AD. The expression of CEmiRs in cluster 4 was significantly downregulated in MCI stages versus healthy controls. In contrast, the expressions of miRNAs in clusters 5 and 6 displayed an increasing trend as AD progressed. Moreover, 11 DECEmiRs displayed opposite expression patterns in the comparisons of MCI vs. healthy controls and that of AD vs. MCI, which are classified into six clusters. MiRNA expression in clusters 7–9 significantly decreased at the stage of MCI compared with that of the healthy control, but showed different degrees of increase at the stage of AD compared with that of MCI. MiRNA expression in clusters 10–12 significantly upregulated at the stage of MCI compared with that of healthy control, but showed different degrees of decline at the stage of AD compared with that of MCI (Figure 2A). These data demonstrate that CEmiRs of AD, MCI, and healthy individuals display distinct expression patterns.

Both microarray and clustering analyses revealed that the majority of DECEmiRs were classified into clusters 1–2, suggesting that DECEmiRs in clusters 1–2 are significantly correlated with AD progression. Importantly, many DECEmiRs in clusters 1–2, including let-7f-5p and miR-485-5p, have been reported with neuroprotective and anti-inflammatory effects in various *in vitro* and *in vivo* models of AD, indicating the potential involvement of DECEmiRs in AD pathogenesis (Han et al., 2018; He et al., 2021). Since miRNAs achieve their biological functions mainly through regulating the expression of their targets, the functional analysis of the predicted targets of DECEmiRs may unveil underlying mechanisms that modulate the progression of AD.

### The predicted targets of differentially expressed CEmiRs were closely linked to Alzheimer’s disease pathogenic processes

**FIGURE 3:**
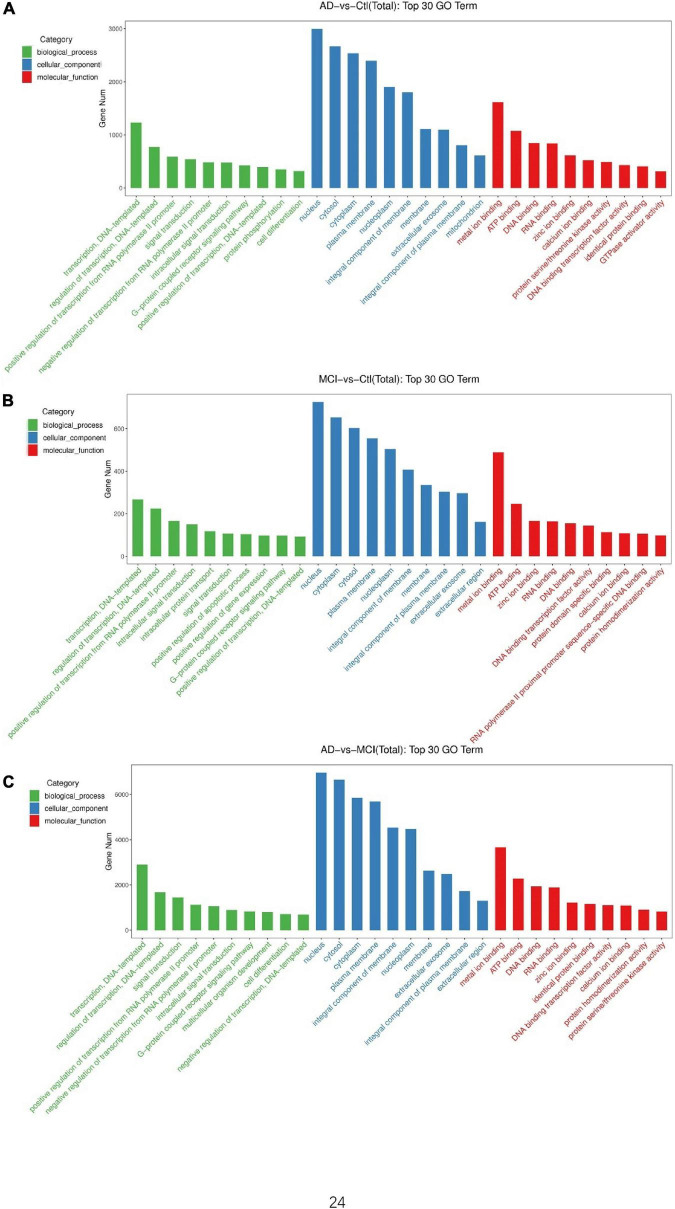
GO analyses of predicted targets of DECEmiRs during AD progression. **(A–C)** The top 10 significant GO terms of biological processes (green bars), cellular components (blue bars), and molecular functions (red bars) of the predicted targets of DECEmiRs in AD vs. Control comparison **(A)**, MCI vs. Control comparison **(B)**, and AD vs. MCI comparison **(C)**. The Y-axis shows the gene numbers and the X-axis shows the GO terms.

To understand the underlying effects of these DECEmiRs on AD pathogenesis, we determined their predicted targets using the miRNA target prediction database Targetscan. Gene ontology (GO) analysis using GO Slim Classification of putative targets of the 29 DECEmiRs in AD vs. Control comparison identified GO terms, such as “G-protein coupled receptor signaling pathway” and “protein phosphorylation” as the top 10 functional clusters of biological processes (Figure 3A). These two GO terms can contribute to pathological processes, including abnormal phosphorylation of tau protein (Chidambaram et al., 2020; Wegmann et al., 2021), deposition of Aβ (Guimaraes et al., 2021), over-activation of microglia (Haque et al., 2018), and dysregulation of calcium homeostasis (Sushma and Mondal, 2019), in AD pathogenesis through various downstream kinases, such as GSK-3β, CDK-5, and ERK signaling cascade. GO analysis of putative targets of the 16 DECEmiRs in MCI vs. Control comparison further identified GO terms such as “positive regulation of apoptotic process” as the top 10 functional clusters, which revealed an apoptosis-mediated neuronal loss in the early onset of AD (Figure 3B). Moreover, analysis of putative targets of the 49 DECEmiRs in AD vs. MCI comparison also identified GO terms such as “G-protein coupled receptor signaling pathway” as the top 10 functional clusters, once again suggesting the involvement of DECEmiRs in disease progression *via* regulating AD-related clusters (Figure 3C).

**FIGURE 4:**
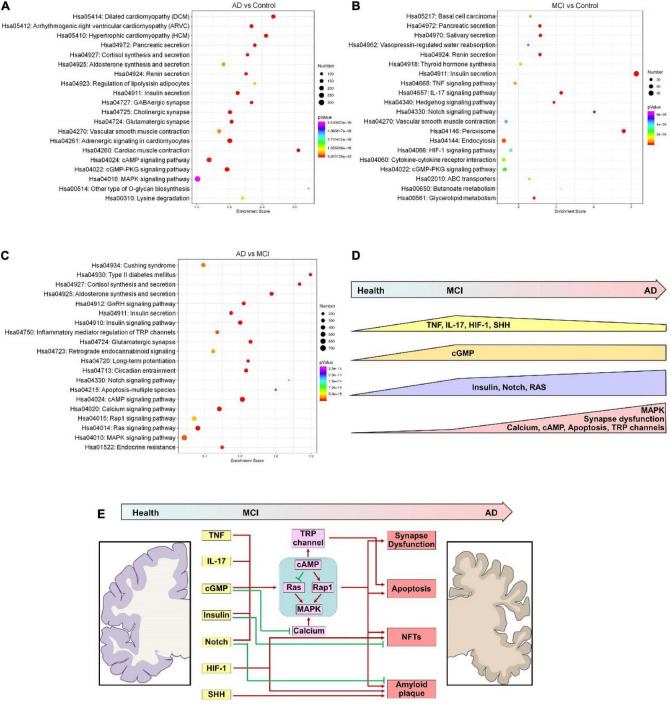
KEGG analyses of predicted targets of DECEmiRs during AD progression. **(A–C)** Top 20 significant signaling pathways of the predicted targets of DECEmiRs in AD vs. Control comparison **(A)**, MCI vs. Control comparison **(B)**, and AD vs. MCI comparison **(C)**. **(D)** The scheme showing the temporal dynamic alterations of signaling pathways regulated by DECEmiRs during AD progression. **(E)** The scheme showing the signaling pathways temporally regulated by DECEmiRs during AD progression and their participation in biological functions.

Next, we performed a KEGG analysis to identify signaling pathways that the predicted targets of these DECEmiRs participated in. Importantly, KEGG analysis revealed that the predicted targets of DECEmiRs in the AD vs. Control comparison were closely linked to “GABAergic synapse,” “Cholinergic synapse,” and “Glutamatergic synapse” pathways, indicating that these DECEmiRs play critical roles in regulating synapse integrity and functions during the disease progression from healthy controls to AD patients (Figures 4A,E and Supplementary Figure 3). Notably, these predicted targets were also strongly linked to “Insulin secretion,” “cAMP signaling,” “cGMP-PKG signaling,” “Renin secretion,” and “MAPK signaling” pathways that play roles in various biological processes, including cell survival, inflammation, and regeneration (Figure 4A and Supplementary Figures 4–6). Furthermore, KEGG analysis of predicted targets of DECEmiRs in the MCI vs. Control comparison showed that these targets were strongly associated with “TNF signaling,” “IL-17 signaling,” and “cytokine-cytokine receptor interaction” pathways, suggesting that these DECEmiRs play pivotal roles in signaling pathways related to inflammation, and that inflammation is an important part of the disease progression from healthy controls to MCI (Figures 4B,E and Supplementary Figure 7). They were also strongly associated with “sonic hedgehog (SHH) signaling,” “Notch signaling,” and “HIF-1 signaling” pathways, suggesting important roles of these pathways in the early onset of AD (Figures 4B,E and Supplementary Figure 8). In addition, analysis of predicted targets of DECEmiRs in the AD vs. MCI comparison revealed that they were closely involved in “Glutamatergic synapse,” “Long-term potentiation,” “Inflammatory mediation of TRP channels,” and “Apoptosis” pathways, implying active participation of these miRNAs in synaptic dysfunction and neuronal loss, which are key pathological features of AD (Figures 4C,E and Supplementary Figures 1, 9–11). Interestingly, these targets were also strongly linked with the “MAPK” signaling pathway and its up- and downstream ones including “Calcium,” “cAMP,” “Rap1,” “Ras,” and “Inflammatory mediation of TRP channels” signaling pathways (Figures 4C,E and Supplementary Figures 5, 6, 9, 10). Given the importance of “MAPK” signaling pathway in the regulation of amyloid plaque and neurofibrillary tangles (NFT) formation, cell death, and neuronal plasticity (D’Mello, 2021), our results suggested that the “MAPK” signaling pathway is a central pathway in the progression of AD from early to late stages.

Together, these results demonstrate a temporally dynamic involvement of signaling pathways underlying important biological functions like inflammation, synaptic functions, and cellular apoptosis during the disease progression from healthy control, to MCI, and to AD (Figures 4D,E).

## Discussion

The potential benefit of the analysis of CEmiRs in the diagnosis of AD has been previously evaluated by numerous researchers (Kumar and Reddy, 2016; Van Giau and An, 2016). Besides being utilized as a diagnostic index, the DECEmiRs confer a great opportunity to us for understanding the pathogenesis of central nervous system disorder, AD, through valuable peripheral samples, which have been neglected. In this study, the altered profiles of CEmiRs among healthy donors and MCI and AD patients have been reported, which can be further classified into 10 clusters. More importantly, bioinformatic analysis of predicted targets of DECEmiRs displayed the complex and precise interactions of signaling pathways that contribute to neuroinflammation, synapse dysfunction, neuroprotection impairment, and neuronal loss during AD progression, suggesting complicated pathogenesis of AD.

To date, numerous studies have reported the altered expression levels of CEmiRs (Liu et al., 2014, 2022; Cheng et al., 2015; Lugli et al., 2015; Gamez-Valero et al., 2019; Dong et al., 2021). We identified multiple DECEmiRs with same expression patterns that were reported by other groups, such as has-let-7-5p, has-miR-106b-5p, has-miR-125b-5p, has-miR-151a-3p, and has-miR-375-3p (Cheng et al., 2015; Gamez-Valero et al., 2019; Dong et al., 2021). Interestingly, the elevated levels of has-miR-125b-5p and has-miR-375-3p within circulating EVs have only been observed in the cohort of MCI and AD patients in Shanghai, China (Dong et al., 2021). This finding may support the potential impact of geographical and environmental factors on AD pathogenesis, a novel research topic that has emerged as a widespread concern (Kessing et al., 2017; Martínez-Solanas et al., 2017; Sun, 2017; Puthusseryppady et al., 2019).

Our informatic analysis of predicted targets of DECEmiRs identified multiple signaling pathways that are activated in the early onset of AD, including TNF, IL-17, HIF-1, and SHH ones. TNF and IL-17 signaling pathways exacerbate neuroinflammation and neurodegeneration in various neurodegenerative diseases (Hennessy et al., 2017; Liu et al., 2019). The activation of inflammatory signaling pathways indicates neuroinflammation as a key early event of AD, matching with our recent finding in an AD animal model (Gao et al., 2019). It dovetails with the well-recognized neuroinflammation hypothesis that proposes neuroinflammation as a driving force for AD (Cameron and Landreth, 2010; Spangenberg and Green, 2017). HIF-1 and SHH signaling pathways also show hyperactivities in the early stage of AD. Both pathways participate in Aβ accumulation and hyperphosphorylation of tau, thus contributing to AD pathogenesis (Li et al., 2021; Wang Y. Y. et al., 2021). Interestingly, HIF-1 and SHH signaling pathways can both be activated by inflammatory signals (Arumugam et al., 2011; McGettrick and O’Neill, 2020), and further promote pro-inflammatory phenotype transition of macrophages/microglia, forming a positive feedback loop to aggravate neuroinflammation (Song et al., 2018; McGettrick and O’Neill, 2020). It is worth noting that, although all these signaling pathways play important roles in neurodegeneration, they are reported to be involved in neuroregeneration and neuroprotection as well (Li et al., 2011; Alam et al., 2016; Pozniak et al., 2016; Sanchez et al., 2018; Wang G. et al., 2021). Thus, the activation of these signaling pathways may reflect protective mechanisms in the early onset of AD.

The bioinformatic analysis identified insulin, Notch, cGMP, and renin secretion signaling pathways whose activities may be altered throughout the whole course of AD. Insulin resistance has been considered as a key culprit in generating the hallmarks of AD arising from neuroinflammation (Akhtar and Sah, 2020; Sedzikowska and Szablewski, 2021). Notch signaling is widely involved in neurovascular damage and amyloid and tau deposition, therefore contributing to AD pathogenesis (Sisodia and St George-Hyslop, 2002; Woo et al., 2009; Kapoor and Nation, 2021). cGMP is a key modulator of synaptic plasticity and long-term potentiation that is instrumental to cognitive functions (Fedele and Ricciarelli, 2021). Renin-dependent synthesis of Ang II has been reported to regulate amyloid plaque formation and memory function mainly through various direct and indirect mechanisms (Gebre et al., 2018; Ribeiro et al., 2020). Hence, our results revealed the close involvement of these signaling pathways in both occurrence and progression of AD.

We also identified multiple signaling pathways that are presumably deregulated at the late stage of AD, including cAMP, calcium, RAS, RAP1, MAPK, TRP channels, apoptosis, and synapse dysfunction. Besides apoptosis and synapse dysfunction which are well-recognized events of AD, the remaining signaling pathways form a complicated network in which MAPK functions as the core node and main downstream effector (Doherty et al., 2000; Waltereit and Weller, 2003; Nussinov et al., 2020). Moreover, MAPK activities can be modulated by deregulated signaling pathways in the early onset of AD, including insulin, Notch, cGMP, and inflammatory ones (Kumphune et al., 2013; Lv et al., 2020; Porcelli et al., 2021; Wei et al., 2021). More importantly, the MAPK signaling pathway has been found to participate in the activation of the apoptotic pathway, formation of NFTs, inhibition of Aβ clearance, and aggravation of excitotoxicity and neuroinflammation (D’Mello, 2021). Therefore, the bioinformatic analysis identified MAPK signaling pathway as a central regulator of AD pathogenesis, indicating the inhibition of MAPK as a potential strategy for AD treatment (Lee and Kim, 2017).

The primary limitation of the present study was the relatively small sample size, as there are only three subjects from each group. Recruiting more patients to validate miRNA microarray results will significantly support our findings, which are currently ongoing. We will report the validation results in future studies. Besides, in the present study, we analyzed the miRNA profiles of circulating EVs that can be released from various organs other than the CNS. Further *in vitro* and *in vivo* studies are necessary to confirm the altered miRNA profiles in brain-derived EVs and the deregulation of key signaling pathways identified in our study.

## Conclusion

In summary, we analyzed the expression profiles of CEmiRs isolated from healthy donors and MCI and AD patients, and classified DECEmiRs into 10 clusters. Inspiringly, the bioinformatic analysis of the predicted targets of DECEmiRs found the temporal deregulation of key signaling pathways that contribute to neuroinflammation, neuronal loss, and synapse dysfunction, which form complicated dynamic signaling networks that control the occurrence and progression of AD. Overall, our study opens a novel window to explore the potential pathogenesis of AD, shedding light on the development of novel therapeutic strategies, including multi-target drug combinations for AD treatment.

## Data availability statement

The datasets presented in this study can be found in online repositories. The names of the repository/repositories and accession number(s) can be found in the article/Supplementary material.

## Ethics statement

The studies involving human participants were reviewed and approved by the Ethics committee of Tongji Hospital affiliated to Tongji University School of Medicine. The patients/participants provided their written informed consent to participate in this study.

## Author contributions

PY, YL, XX, and JCZ designed the experiments. PY, LD, JZ, XQ, and YZ performed the experiments. YW, PY, and XX analyzed the data. YW and XX prepared the manuscript. All authors read and approved the final manuscript.

## Footnotes

1
www.targetscan.org

2
https://david.ncifcrf.gov/

3
https://www.mirbase.org/
