# Supplementary material for: Circulating extracellular vesicle-containing microRNAs reveal potential pathogenesis of Alzheimer’s disease
Source: Front Cell Neurosci. 2022 Oct 20;16:955511. doi: 10.3389/fncel.2022.955511 (PMC9630335; doi:10.3389/fncel.2022.955511)
Supplement: Supplementary file 1 [file Data_Sheet_1.docx]

**Supporting Information**

**Circulating extracellular vesicle-containing microRNAs reveal potential pathogenesis of Alzheimer's disease**

Yi Wang^1ǂ^, Ping Yuan^2ǂ^, Lu Ding^3ǂ^, Jie Zhu^4^, Xinrui Qi^3^, Yanyan Zhang^4^, Yunxia Li^5*^, Xiaohuan Xia^3,6,7*^, Jialin C. Zheng^3,6,7,8*^

^1^Translational Research Center, Shanghai Yangzhi Rehabilitation Hospital affiliated to Tongji University School of Medicine, Shanghai 201613, China. ^2^Department of Cardio-Pulmonary Circulation, Shanghai Pulmonary Hospital, Tongji University, School of Medicine, Shanghai 200433, China. ^3^Center for Translational Neurodegeneration and Regenerative Therapy, Tongji Hospital affiliated to Tongji University School of Medicine, Shanghai 200072, China. ^4^Center for Translational Neurodegeneration and Regenerative Therapy, Shanghai Tenth People’s Hospital affiliated to Tongji University School of Medicine, Shanghai 200072, China. ^5^Department of Neurology, Tongji Hospital, School of Medicine, Tongji University, Shanghai 200092, China. ^6^Translational Research Institute of Brain and Brain-Like Intelligence, Shanghai Fourth People's Hospital affiliated to Tongji University School of Medicine, Shanghai 200434, China. ^7^Shanghai Frontiers Science Center of Nanocatalytic Medicine, Tongji University, Shanghai 200331, China.^8^Collaborative Innovation Center for Brain Science, Tongji University, Shanghai 200092, China.

**Contents**

Supplemental Table 1

Supplemental Figure 1

Supplemental Figure 2

Supplemental Figure 3

Supplemental Figure 4

Supplemental Figure 5

Supplemental Figure 6

Supplemental Figure 7

Supplemental Figure 8

Supplemental Figure 9

Supplemental Figure 10

Supplemental Figure 11

| **Variable** | **Control** | **MCI** | **AD** |
| --- | --- | --- | --- |
| No. of subjects | 3 | 3 | 3 |
| Age (year) | 70.0 ± 7.1 | 67.3 ± 4.7 | 69.2 ± 5.9 |
| BMI (kg/m^2^) | 21.8 ± 1.9 | 25.3 ± 1.3 | 24.8 ± 0.6 |
| Heart rate (per min) | 81.3 ± 3.2 | 79.0 ± 5.6 | 82.3 ± 14.0 |
| Systolic blood pressure (mmHg) | 127.7 ± 7.1 | 139.3 ± 17.9 | 138.3 ± 23.0 |
| Diastolic blood pressure (mmHg) | 85.0 ± 7.8 | 87.3 ± 7.6 | 96 ± 14.3 |

**Supplemental Table 1.** Baseline characteristics of study participants

**
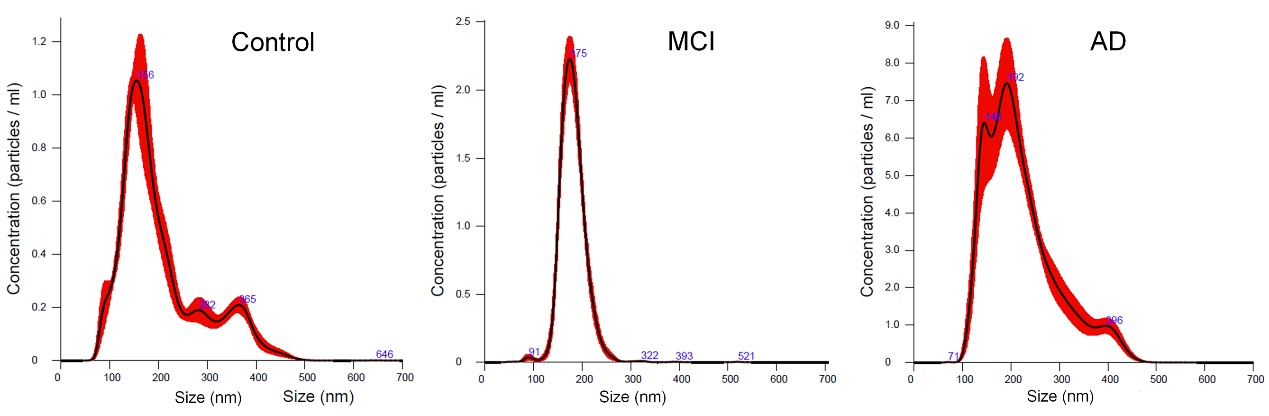
Supplementary Figure 1. NTA analysis of EVs.**

Particle-size distribution and concentration of EVs among groups were determined by NTA.


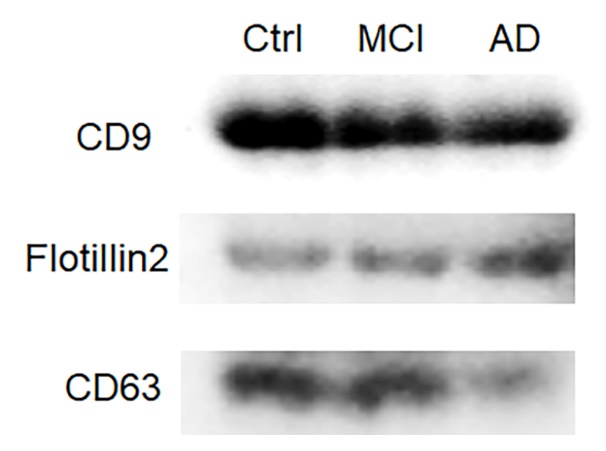


**Supplementary Figure 2. Western blotting analysis of EVs.**

The expression of EV positive markers CD9, Flotillin2, and CD63 in protein lysates of EVs were determined by western blotting.

**
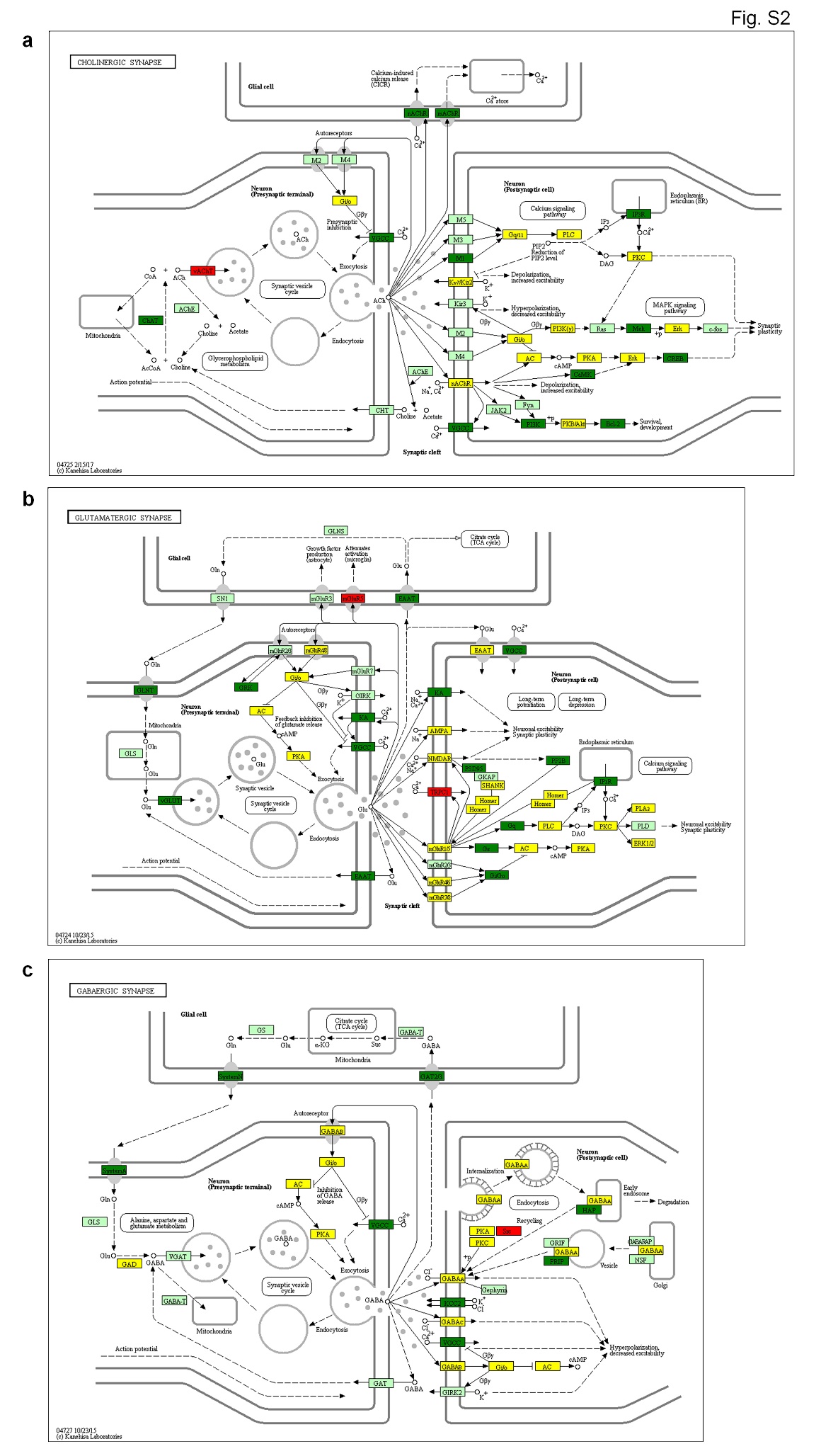
**

**Supplementary Figure 3.** The schematic picture of cholinergic synapse (**A**), glutamatergic synapse (**B**), and GABAergic synapse (**C**) pathways.

**
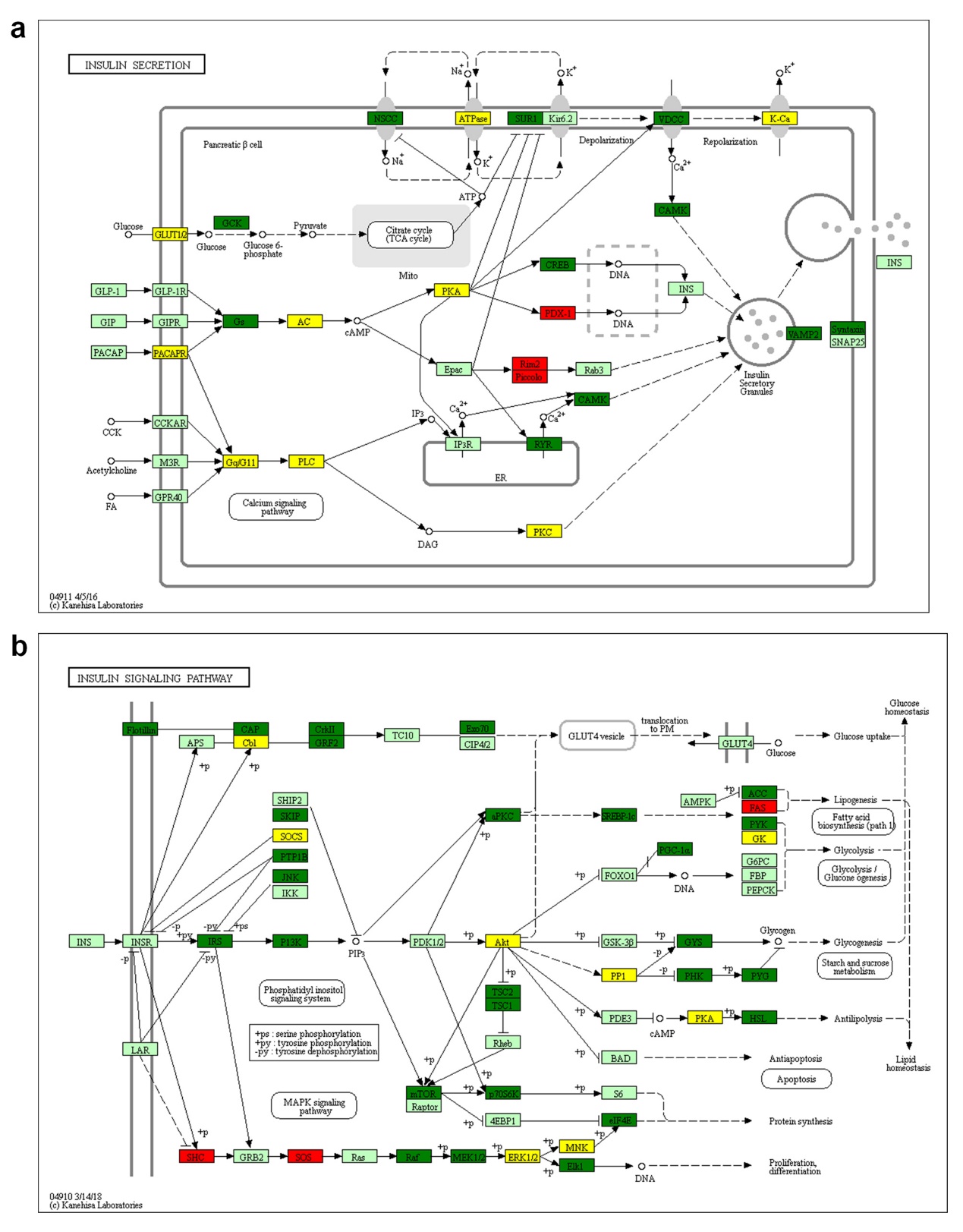
**

**Supplementary Figure 4.** The schematic picture of insulin secretion (**A**) and insulin signaling pathways (**B**).

**
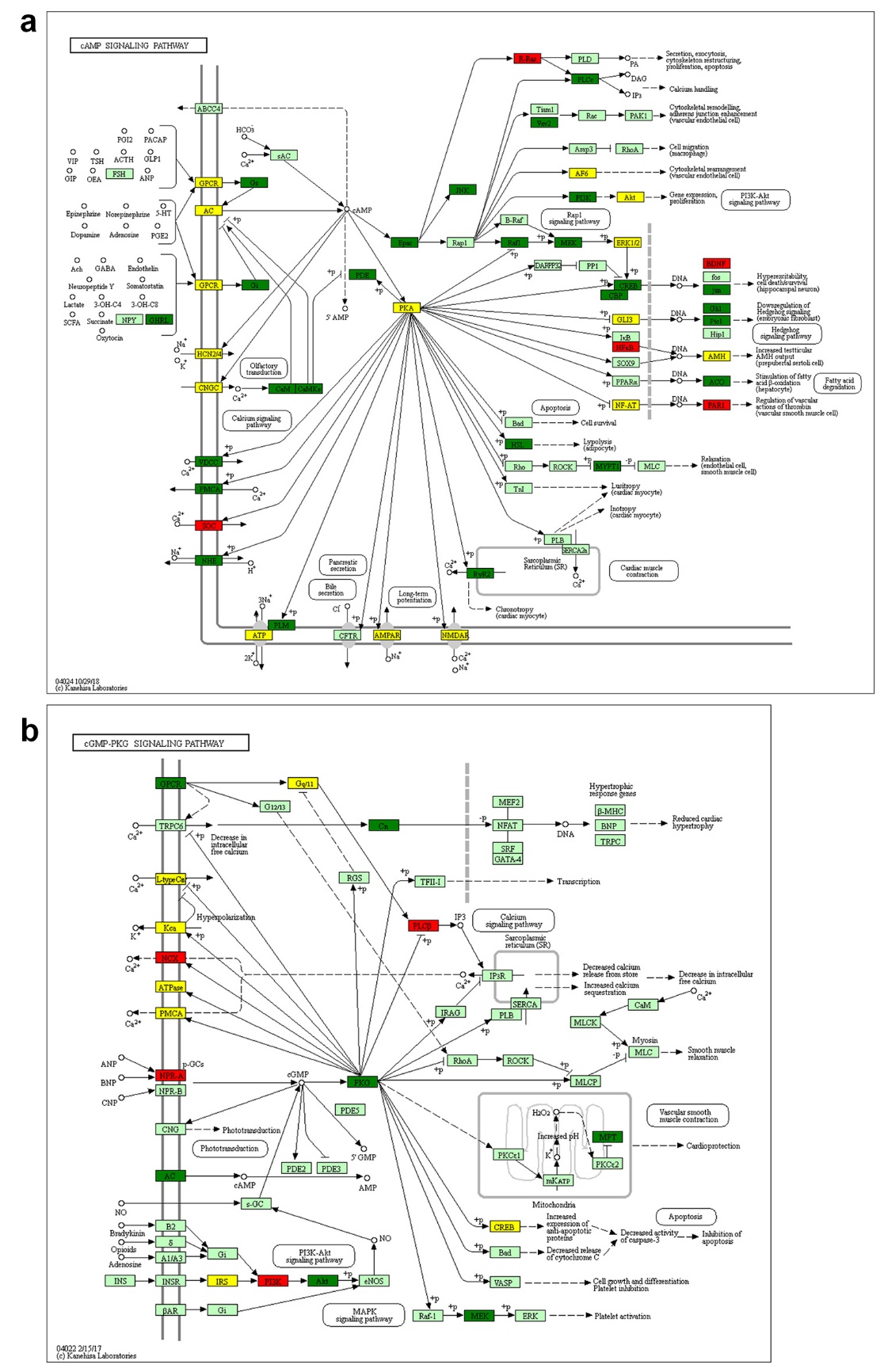
**

**Supplementary Figure 5.** The schematic picture of cAMP (**A**) and cGMP (**B**) signaling pathways.

**
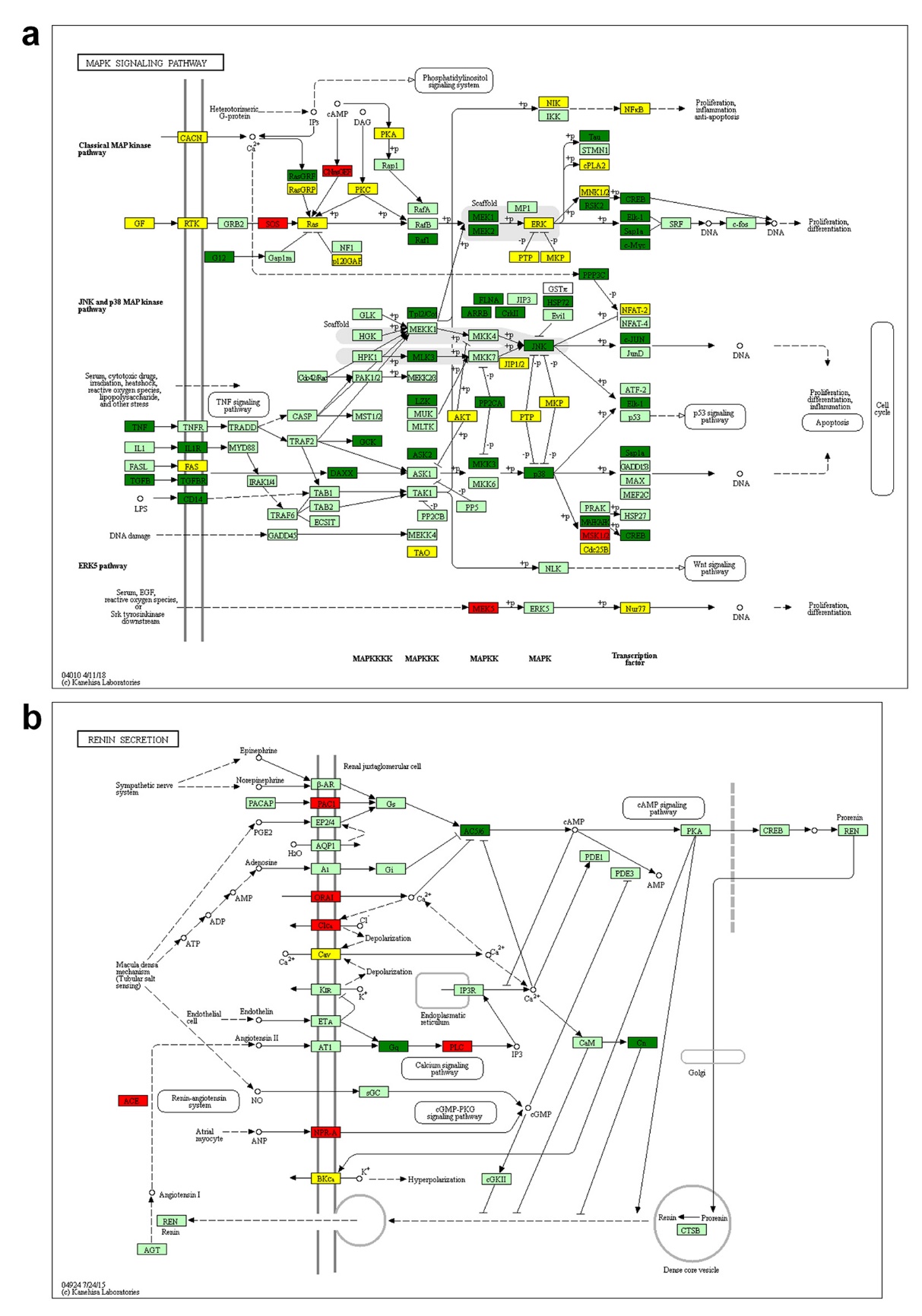
**

**Supplementary Figure 6.** The schematic picture of MAPK signaling (**A**) and Renin secretion (**B**) pathways.

**
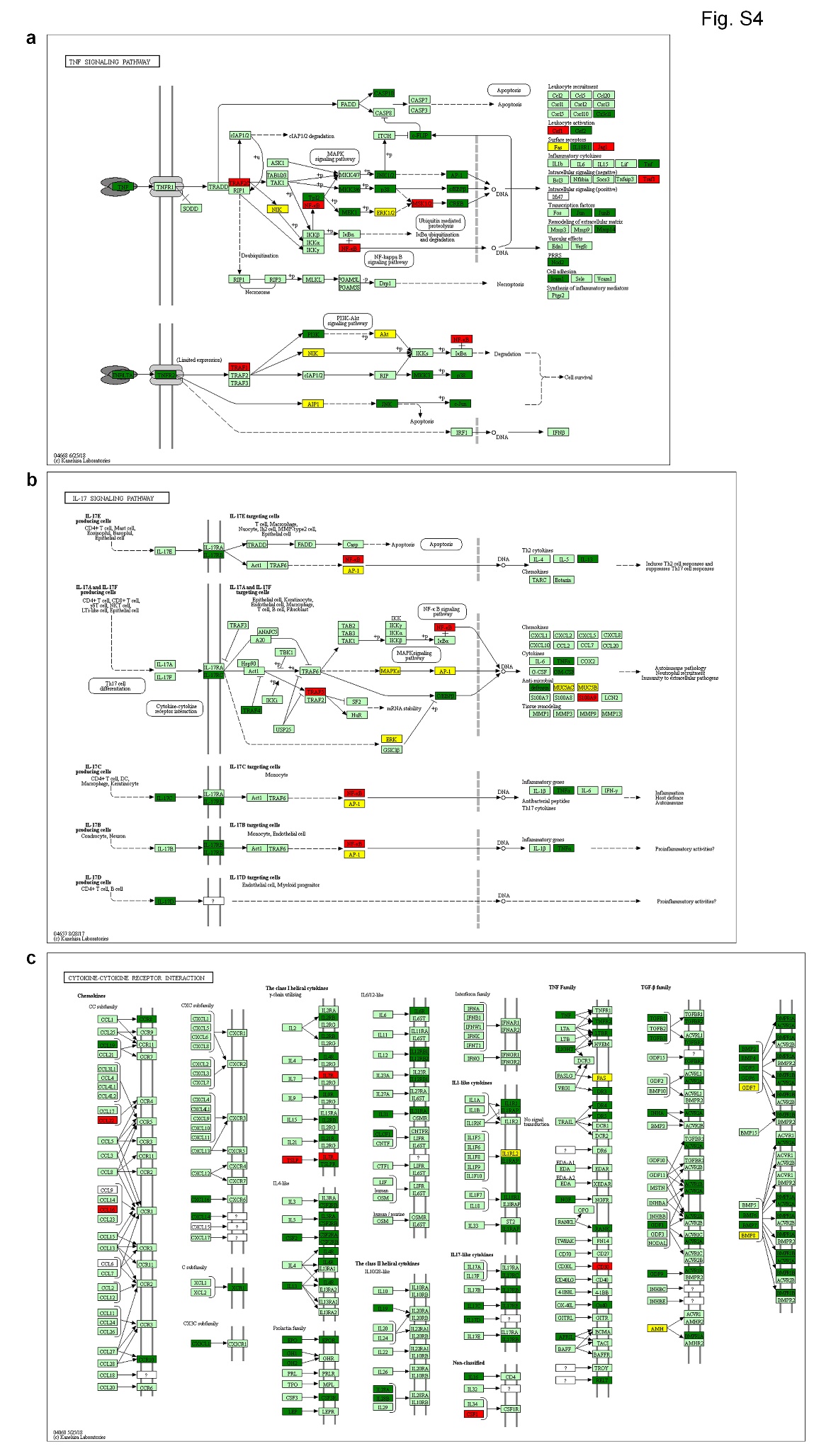
Supplementary Figure 7.** The schematic picture of TNF signaling (**A**), IL-17 signaling (**B**), and cytokine-cytokine receptor interaction (**C**) pathways.

**
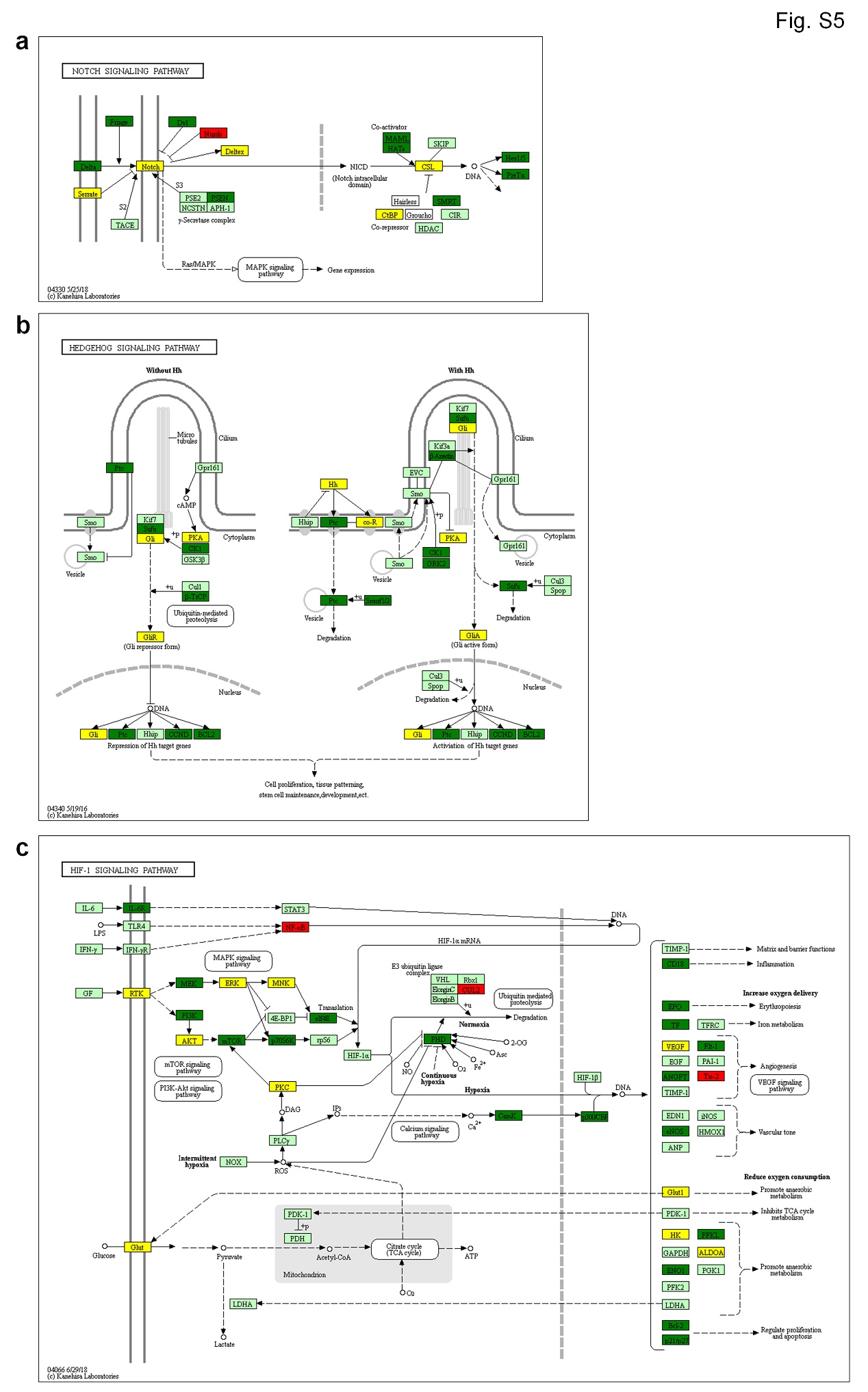
Supplementary Figure 8.** The schematic picture of Notch (**A**), sonic hedgehog (**B**), and HIF-1 (**C**) signaling pathways.

**
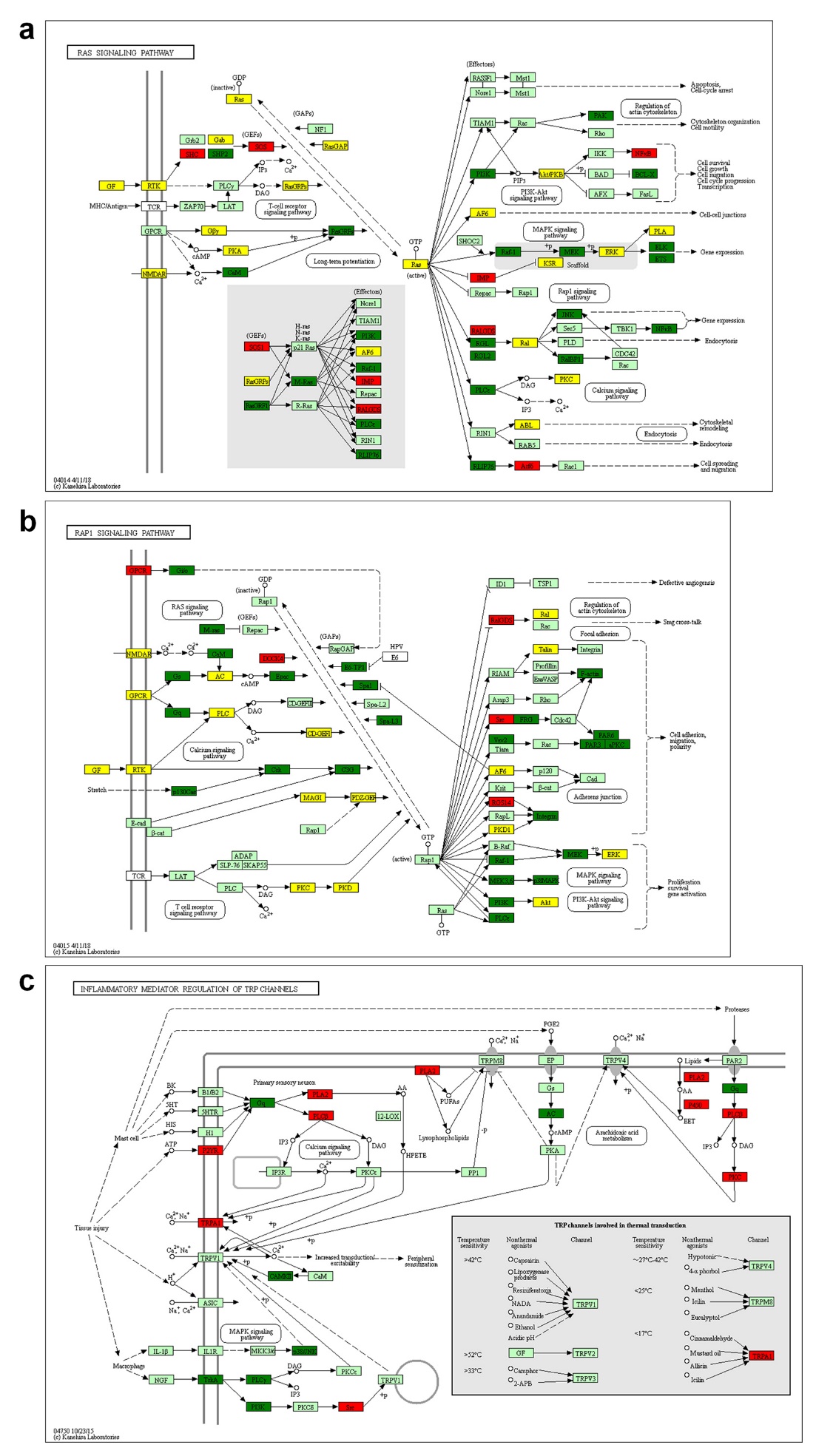
**

**Supplementary Figure 9.** The schematic picture of Ras (**A**), Rap1 (**B**), and Inflammatory mediator regulation of TRP channels (**C**) signaling pathways.

**
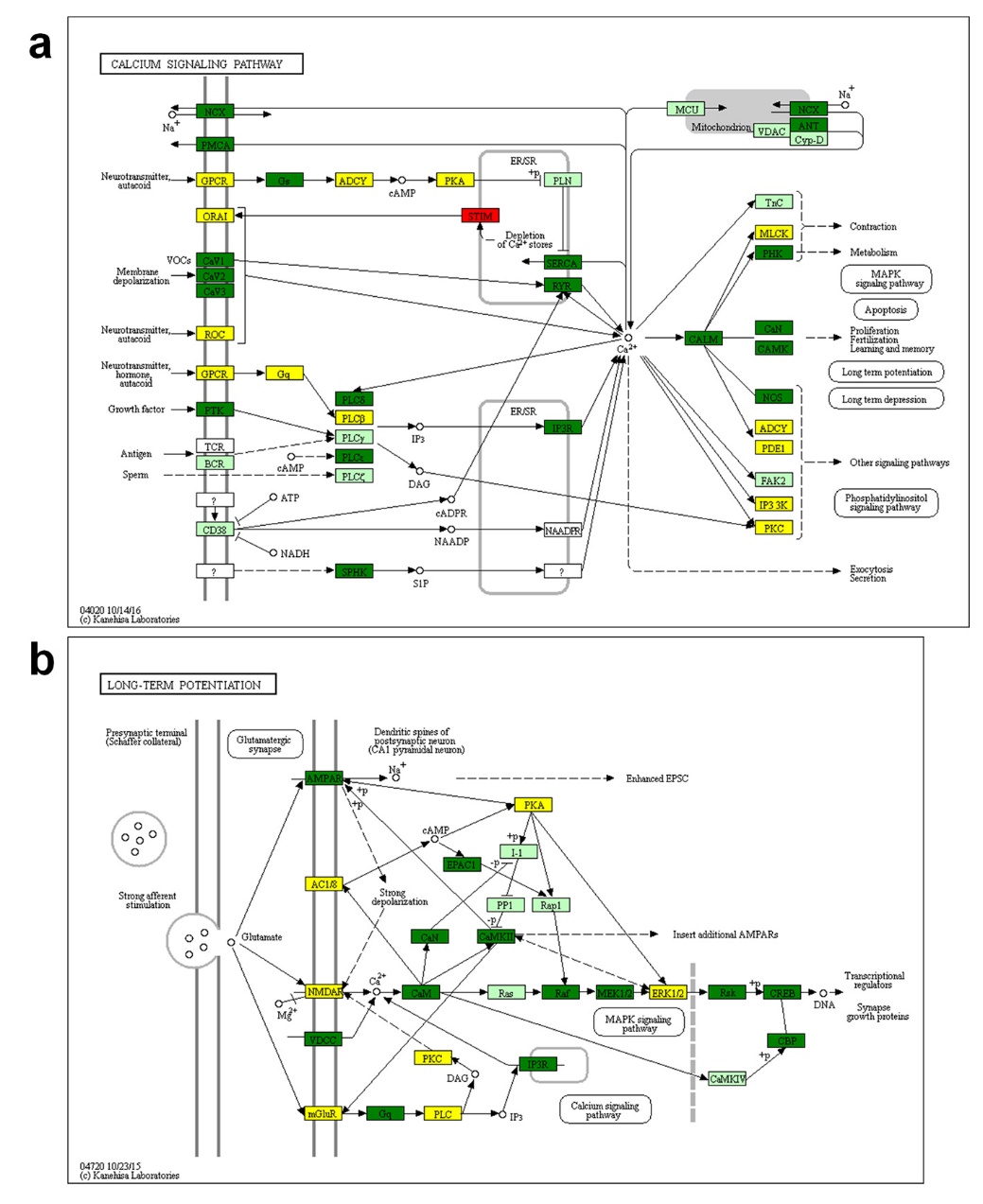
Supplementary Figure 10.** The schematic picture of calcium signaling (**A**), and long-term potentiation (**B**) pathways.

**
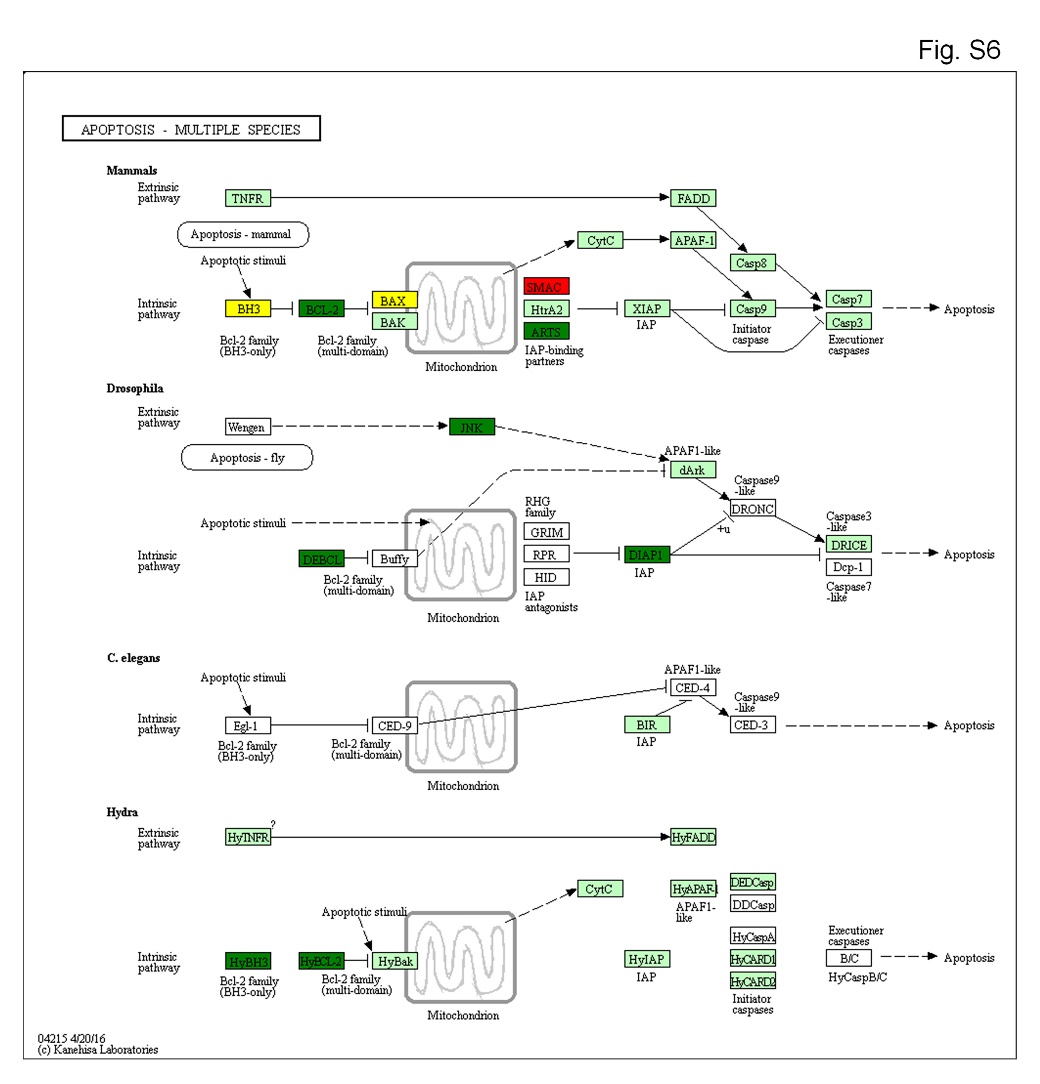
Supplementary Figure 11.** The schematic picture of apoptotic pathways in multiple species.
